# Differential gene expression in skin RNA of horses affected with degenerative suspensory ligament desmitis

**DOI:** 10.1186/s13018-020-01994-y

**Published:** 2020-10-07

**Authors:** Abigail Haythorn, Madeline Young, James Stanton, Jian Zhang, P. O. E. Mueller, Jaroslava Halper

**Affiliations:** 1 Department of Pathology, College of Veterinary Medicine, The University of Georgia, Athens, GA 30602 USA; 2 Department of Large Animal Medicine, College of Veterinary Medicine, The University of Georgia, Athens, GA 30602 USA; 3 AU/UGA Medical Partnership, The University of Georgia, Athens, GA 30602 USA

**Keywords:** Equine degenerative suspensory ligament desmitis, Next generation sequencing, Differential expression of genes, BMP2, Proteoglycans, Collagens, Keratins

## Abstract

**Background:**

Equine degenerative suspensory ligament desmitis (DSLD) is a systemic connective tissue disorder first identified in Peruvian Paso horses but afflicting other horse breeds as well. Inappropriate accumulation of proteoglycans in connective tissues, most prominently in tendons and ligaments, leads to progressive and debilitating lameness and pain. It is largely unknown what drives the overproduction of proteoglycans, but our previous studies suggest involvement of bone morphogenetic protein 2 (BMP2), a member of the transforming growth factor-β (TGFβ) family, impacting synthesis of proteoglycans. To identify potential players in pathogenesis of DSLD a new approach utilizing next generation sequencing was undertaken.

**Methods:**

Next generation sequencing was performed using RNA extracted from skin biopsies of six control Peruvian Pasos and six horses with DSLD (4 Peruvian Pasos and 2 warmbloods). The CuffDiff result sets were validated with algorithms used to run them. This was based on the determined false discovery rates derived from the *P* values adjusted for multiple testing for any given result.

**Results:**

Bioinformatics analysis of transcriptomes revealed differential expression of over 1500 genes, including increased expression of genes for several growth factors (most prominently BMP2, FGF5, CTGF, many members of the EGF family), and mediators of signaling (Fos, Myc, MAPK system), and keratins. Two genes encoding for enzymes involved in synthesis of hyaluronan were also overexpressed. Gene expression was decreased for protein cores of many proteoglycans, several growth factors, most collagens, and many peptides with immune function.

**Conclusions:**

The overexpression of *BMP2* correlates well with our previous data. However, the decrease in expression of numerous proteoglycans was unexpected. A mutation in a gene of a less characterized proteoglycan and/or glycosyltransferase with subsequent increased production of hyaluronan and/or a proteoglycan(s) undetected in our study could account for the systemic proteoglycan deposition. Decreased collagen gene expression indicates abnormal connective tissue metabolism. The increased expression of keratin genes and *FGF5* supports reports of skin abnormalities in DSLD. Underexpression of immune function genes corresponds with lack of inflammation in DSLD tissues. Finally, though the proteoglycan and/or glycosaminoglycan abundant in DSLD has not been identified, we validated our previous data, including overexpression of *BMP2*, and systemic nature of DSLD due to disturbed metabolism of the extracellular matrix.

## Background

Equine degenerative suspensory ligament desmitis (DSLD) is a debilitating systemic disorder afflicting primarily the tendons and ligaments of the distal limb horses, and also other systems with high content of certain components of extracellular matrix, such as the large vessels and sclerae [1]. As the condition worsens, abnormalities in the biomechanical and structural integrity of the tendons and ligaments lead to characteristic dropping of the fetlock and pastern, progressive and debilitating bilateral and quadrilateral lameness, and enlargement with multifocal hypoechoic lesions of affected tendons and ligaments on ultrasonographic examination [1]. All too often to the sequela is humane euthanasia due to the progressive lameness and pain [1, 2].

A hereditary pattern of DSLD has been observed, especially in Peruvian Paso horses. In addition, other breeds, such as warmbloods and quarter horses, are affected as well [1, 3]. The diagnosis of DSLD is based on signalment and history, physical and ultrasound examination, and, in selected cases, subjective evaluation of a biopsy of the nuchal ligament (Halper and Mueller, unpublished data). However, presently, post-mortem and histopathological examinations are the only methods of providing a definitive diagnosis [1, 4]. Originally, DSLD was considered to be the result of a primary collagen dysfunction limited to suspensory ligaments of the lower extremities [2, 5]. Our lab has demonstrated that DSLD is a systemic disorder with the hallmark of an excessive buildup of proteoglycans in equine organs and tissues with high content of extracellular matrix [1]. In most cases, characteristic changes consisting of pools or network of proteoglycans disrupting collagen scaffolding are found between fibers or bundles of collagens, or replacing collagen and other structures completely. The largest amounts of proteoglycans are present in affected tendons and ligaments. Clinically healthy tissues from DSLD-affected horses, including tendons, aortas, coronary arteries, and sclerae among other organs, contain abnormal accumulation of extracellular proteoglycans as well [1]. In addition to the more classic clinical symptoms, skin abnormalities (loose skin and white hair spots) have also been observed (personal communications with horse owners). Overall, only handful descriptions of clinical and histopathologic DSLD have been published, with our work appearing to be the most comprehensive [1].

Unfortunately, there is no cure or treatment for DSLD, only palliative and supportive treatment (NSAIDs, controlled exercise, and supportive shoeing) [6]. Additionally, the pathogenesis has not been fully characterized, though our previous data suggest defect(s) in processing and/or metabolism of proteoglycans. We have demonstrated that the dermatan sulfate chains have been replaced at least partially with chondroitin sulfate indicating a defect in proper glycosylation of decorin and/or of other proteoglycans [4]. Plaas et al. have uncovered altered metabolism of aggrecan [7]. More recently, we have identified increased presence of bone morphogenetic protein 2 (BMP2) in active cellular lesions in DSLD-affected tendons, indicating that stimulation by TGFβ-related growth factors may play a role in DSLD pathogenesis [8]. Previous attempts aimed at identification of a specific genetic defect responsible for DSLD have been unsuccessful (personal communication with other investigators). In this study, we report the results of next generation sequencing (NGS) of RNA samples obtained from Peruvian Pasos and warmbloods, both healthy and afflicted with DSLD to determine which changes in equine transcriptomes might contribute to better diagnosis of DSLD and to better understanding of its pathogenesis.

The search for a reliable, safe, and palatable antemortem test to confirm the diagnosis of DSLD has been challenging. The tendons and ligaments of the equine distal limb have a small cross-sectional area and are under maximal stress and strain during work and exercise [9, 10]; therefore, even the smallest excisional biopsy puts the horse at an unacceptable risk of future tendon injury and/or catastrophic failure, making the veterinarian reluctant to recommend the biopsy procedure and the horse owner even less willing to allow it. We have previously used subjective histologic evaluation of the nuchal ligament in an attempt to identify affected horses. This method lacks the specificity necessary to make an accurate and reliable diagnosis (Halper and Mueller, unpublished data).

In this study, we have chosen to use skin as the source of RNA and subsequent sequencing in an effort to develop a more specific and safer antemortem diagnosis for DSLD. Skin biopsies are minimally invasive, heal quickly with minimal adverse cosmetic effects, and as such are more acceptable to the veterinarian and horse owner. The technique also eliminates the concerns and morbidity associated with direct biopsy of supporting tendons and ligaments.

In addition, tendons affected with DSLD have many areas interlaced or replaced with either acellular masses of proteoglycans or with metaplastic cartilage which would lead to insufficient RNA amount and introduce further variabilities into gene expression assessment. Therefore, skin because of its accessibility and relative ease of obtaining the biopsy is an excellent tissue to be used for diagnostic purposes.

Next generation sequencing, or in this case RNA-seq, represents a high-yield approach for transcriptomics where not only transcript sequences are obtained but measurements of levels of individual transcripts became possible as well [11]. Thus, this methodology may provide useful information on gene expression in a variety of tissues [11, 12]. Though skin involvement appears to be less significant in DSLD, the differential expression of genes in this organ informs on the systemic nature of DSLD, not limited to suspensory ligaments and tendons as postulated in the past [2]. We hypothesize that histologic and molecular biological examination of skin from affected DSLD horses will provide a safe, specific, and accurate anti-mortem test for DSLD.

## Methods

### Experimental subjects

All participating horses came from private sources and were either donated to the University of Georgia or underwent skin biopsies with full consent of owners. Skin samples were obtained from six control and six DSLD-affected horses and used to extract RNA for subsequent NGS. All six control and four DSLD-affected horses were Peruvian Pasos. The two remaining DSLD-affected horses were warmbloods. Table 1 shows the diagnosis of DSLD was based on clinical examination (which included physical examination, and in some cases ultrasound) and on necropsy in the other 50%. Both sexes were represented, and their age ranged from 3 years to mid-30’s (Table 1).

**Table 1:** Experimental subjects

Sample ID	Breed	Age sex	Diagnostics
CTL1	PP	13 M	Clinical
CTL2	PP	29 F	Clinical
CTL3	PP	31 F	Necropsy
CTL4	PP	31 F	Necropsy
CTL5	PP	32 F	Necropsy
CTL6	PP	3 F	Clinical
DSLD1	PP	15 M	Clinical
DSLD2	PP	Mid 20s F	Necropsy
DSLD3	PP	5 F	Necropsy
DSLD4	WB	18 M	Clinical
DSLD5	PP	20 M	Necropsy
DSLD6	WB	> 35 M	Clinical

### Skin biopsy

The biopsy procedure described here pertains only to six biopsies performed at the University of Georgia. Remaining biopsies were supplied by participating veterinarians or horse owners. A protocol standard at our Veterinary Teaching Hospital was followed: 1 h before the biopsy, horses were given an intramuscular injection of procaine penicillin G (22,000 IU/kg) and intravenous dose of phenylbutazone (4.4 mg/kg) before being placed in standing docks. A 15 cm × 15 cm mid cervical area was clipped and aseptically prepared with chlorhexidine and alcohol (3 ×) before sedation with intravenous administration of acetylpromazine (0.02 mg/kg), detomidine HCl (20 μg/kg), and butorphanol tartrate (0.02 mg/kg). This was followed by local anesthesia (subcutaneous injection of 20 ml 2% lidocaine hydrochloride). Standard single use skin punches (6 or 7 mm in diameter) were utilized to obtain 2–3 full thickness biopsies from the neck skin of three control and three DSLD-affected horses who underwent clinical examination. A small excisional biopsy was performed on the neck of three control and three DSLD-affected Peruvian Paso horses who were donated and were euthanized after skin excision. Each biopsy site was closed by one or two interrupted suture of 2-0 Prolene.

Necropsy was performed on all six euthanized horses; however, the timing and logistics did not allow for quick removal of samples from tendons and subsequent extraction of intact RNA.

Animal use protocol for skin biopsies and necropsy protocol was approved by the IACUC at the University of Georgia.

### RNA extraction and preparation

Obtained skin samples were immediately immersed in RNALater solution (Invitrogen, Thermo Fisher Scientific, Corp., Carlsbad, CA) to preserve RNA and were delivered to Halper’s lab where RNA extraction was done within a day or two using the RNeasy mini kit as recommended by the manufacturer (Qiagen, Germantown, MD, USA). After extraction and purification, total RNA samples were frozen at − 80 °C for 1–6 months (one sample was frozen for 1 year) before submission for NGS and initial bioinformatics to the Georgia Genomics and Bioinformatics Core (GGBC) at the University of Georgia.

### cDNA library preparation, next generation sequencing, and creation of analysis workflow, statistics

**Fig. 1:**
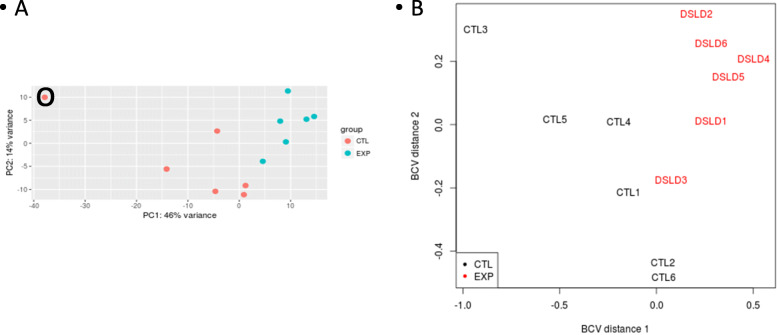
Principal component analysis: full matrix: PCA analyses of both the full and filtered matrices showed that the samples from control and experimental grouped together well with expected variance among individuals. **a** This analysis was run using the R-code in file DESeq2. Outlier CTL3 is marked by a black circle. **b** Using the R-code in file EdgeR this analysis showed very similar results

Stranded sequence libraries for equine control and DSLD cell populations were prepared from total RNA as recommended by the manufacturer of the KAPA Stranded mRNA-Seq kit (Kappa Biosystems, Wilmington, MA, USA). Paired-end 75 bp reads (PE-75) were generated at GGBC on an Illumina NextSeq 500 instrument using a high output flow cell (San Diego, CA, USA). Average library size exceeded 40 million paired-end reads. Read quality of raw and trimmed RNA-Seq data was assessed using FastQC (Babraham Bioinformatics, Babraham Institute, Cambridge, UK), and quality trimming was performed using the Trimmomatic software [13]. Reads whose trimmed length fell below 50 bases were discarded. Trimmed reads were aligned to the *E. caballus* genome (EquCab3.0, NCBI Accession GCF_002863925.1) (Table 2). Read alignment to the reference genome was done usingTopHat2 [14] run at default settings. Cufflinks [14] was employed to assemble transcripts, and CuffDiff (a component of the Cufflinks package) was used to determine and quantify differential gene expression and fold expression differences after FPKM normalization [13]. Two other systems, DESeq2_DEG and edgeR, were used but only in the initial analysis as there was a significant overlap among the three programs, and Cufflinks is preferred by most scientists in the field. Each of the result sets was statistically validated with the algorithms used to run them, i.e., CuffDiff, DESeq2, and edgeR. *P* values were adjusted for multiple testing for determination of false discovery rates (FDR) using the Benjamini-Hochberg correction [15, 16]. Principal component analysis (PCA) of both full matrices using the R-code in files DESeq2 and EdgeR was utilized to show separation between control and experimental groups well with expected variance among individuals (Fig. 1). PCA showed that one control sample (CTL3) was an extreme outlier (Fig. 1a). Differentially expressed genes were identified based on a log fold change of 2.

**Table 2:** Source of equine genome information

NCBI accession	Genome information	Renamed file	Bow tie2 index
GCF_002863925.1_EquCab3.0_genomic.fna	E. caballus assembly version 3.0	Ecab.fa	Ecab
GCF_002863925.1_EquCab3.0_genomic.gff	E. caballus assembly version 3.1	Ecab.gff	Ecab

All the raw data are available in the NCBI Sequence Read Archive (accession number: PRJNA544650). The Cufflinks data are available in the Supplemental Data.

### Functional classification of differentially expressed genes

Differentially expressed genes (DEGs) were identified based either on log fold change of 2 or on cutoff of FDR less than 0.05 (significant *q* value) [17]. Data analysis of genes with significant *q* value was done using the Panther (Protein ANalysis THrough Evolutionary Relationships) Classification System, a database organizing sequences of genes/proteins into families and their functionally related subfamilies. This system is used to classify and identify the function of gene product/transcripts in a variety of biological processes, such as signaling pathways [18, 19]. The graphs in Figs. 3, 4, 5, 6, 7, and 8 show genes, clustered by PANTHER analysis.

## Results

### Experimental subjects

Skin samples for RNA extraction were obtained from twelve horses, 10 Peruvian Pasos and two warmbloods (Table 1). We opted to use skin as a source of RNA for several reasons. Though tendons and ligaments are the most severely affected tissues in DSLD, it is not practical and rather harmful to attempt to biopsy tendons of any horses, healthy or affected. Logistically, it turned out we were not able to obtain tendon tissue providing enough RNA either because of the time lag between euthanasia and opportunity to harvest tendon and because of low RNA yield in tendons with very high proteoglycan deposits. As DSLD is a systemic disease affecting connective tissues of many organs, including skin, biopsies of skin provide a safer and much simpler way of obtaining tissue samples.

### Bioinformatics analysis

The pooled libraries generated from the 12 individual RNA samples were sequenced on the Illumina’s NextSeq500 platform using the PE75 read length protocol. The read yield was approximately 40 million paired-end reads per sample with no quality trim of the raw data necessary. TopHat workflow analysis identified splice junctions and generated read alignments for each of the 12 samples. Principal component analysis (PCA) of both full matrices using the R-code in files DESeq2 and EdgeR showed that the samples from control and experimental grouped together well with expected variance among individuals (Fig. 1). PCA showed that one control sample (CTL3) was an extreme outlier (Fig. 1a). Each of the result sets was statistically validated with the algorithms used to run them, i.e., CuffDiff (a component of the Cufflinks package—see below), DESeq2, and EdgeR, and are ultimately based on the determined false discovery rates (FDR), which are derived from the *P* values adjusted for multiple testing for any given result. It was the Cufflinks package utilized by us for functional assessment because of its ease of use by many other investigators. Using CuffDiff, 32,823 genes corresponding to 80,518 transcripts were detected.

**Fig. 2:**
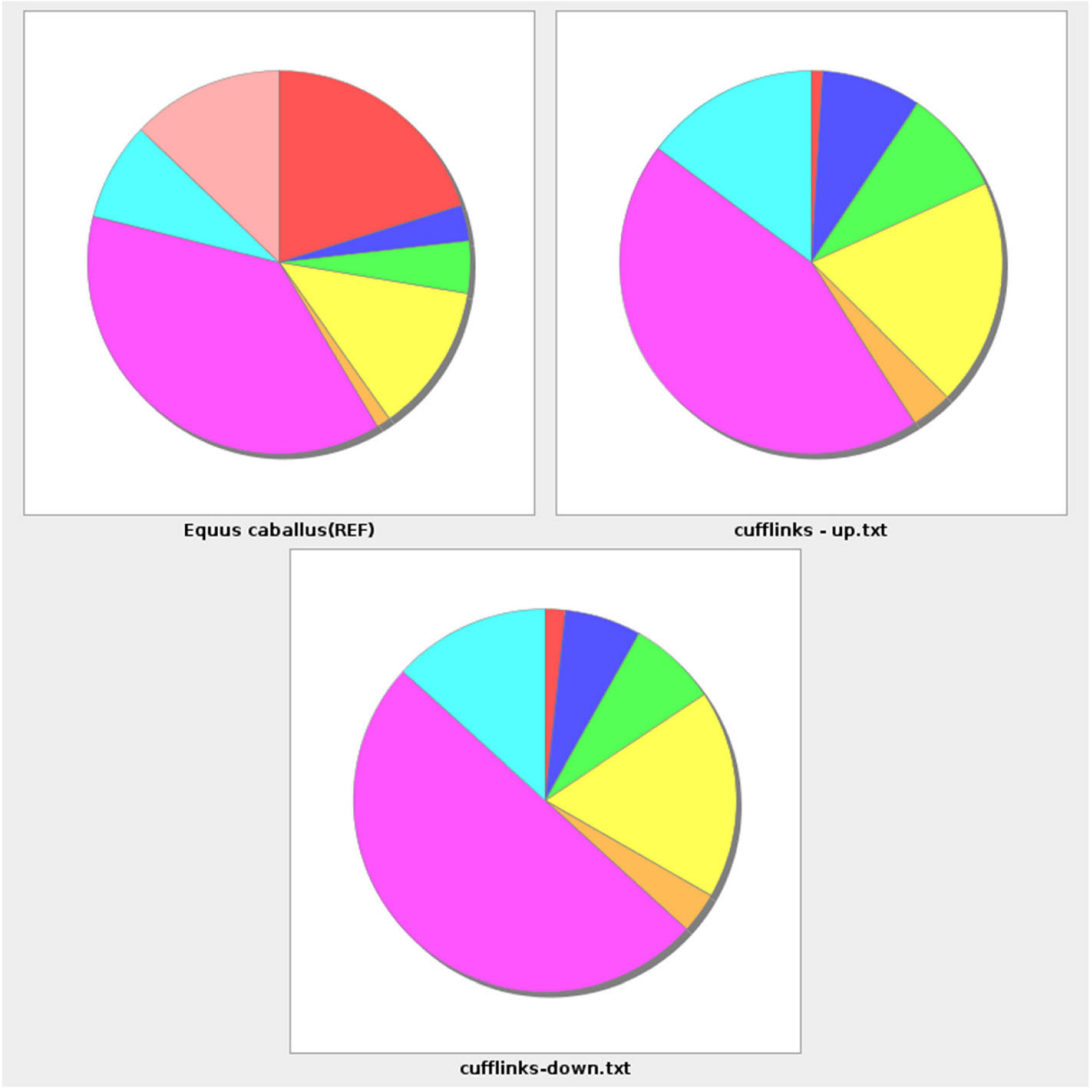
PANTHER GO analysis of DEG of (slim) molecular functions in DSLD: The pie chart shows changes in gene expression identified by Cufflinks against genes in the Equus caballus genome database. The following gene categories were evaluated: red, RNA binding (GO:0003723); blue, calcium ion binding (GO:0005509); green, metal ion binding (GO:0046872); yellow, molecular function regulator (GO:0098772); orange, protease binding (GO:0002020); violet, protein binding (GO:0005515); light blue, signaling receptor binding (GO:0005102); pink, single-stranded RNA binding(GO:0003727)

Further functional Cufflinks evaluation identified 1567 differentially expressed genes (DEG) at an FDR cutoff ≤ 0.05. 1332 genes were annotated, and 708 genes also had at least a 2-fold change in expression. Of the 1567 genes, 617 were overexpressed genes and 950 underexpressed genes in DSLD horses (in comparison to genes expressed in control horses, see Supplemental data). Using the Panther GO system, DEGs were summarized in four categories: cellular components, protein classes, molecular functions, and biological processes. Panther analysis of DEG participating in molecular functions in DSLD reveals substantial changes (both up and down) in several categories of genes: those responsible for protein and receptor binding, and for regulators of molecular function (Fig. 2).

As very little is known about the pathogenesis of DSLD and involvement of specific genes and proteins in its development and course, we focused our attention on expression of genes of known significance in pathways important for physiology and metabolism of connective tissues and of extracellular matrix (ECM) in particular. Genes encoding for unknown or at least unnamed proteins were omitted from consideration.

### Growth factors and signaling pathways

**Fig. 3:**
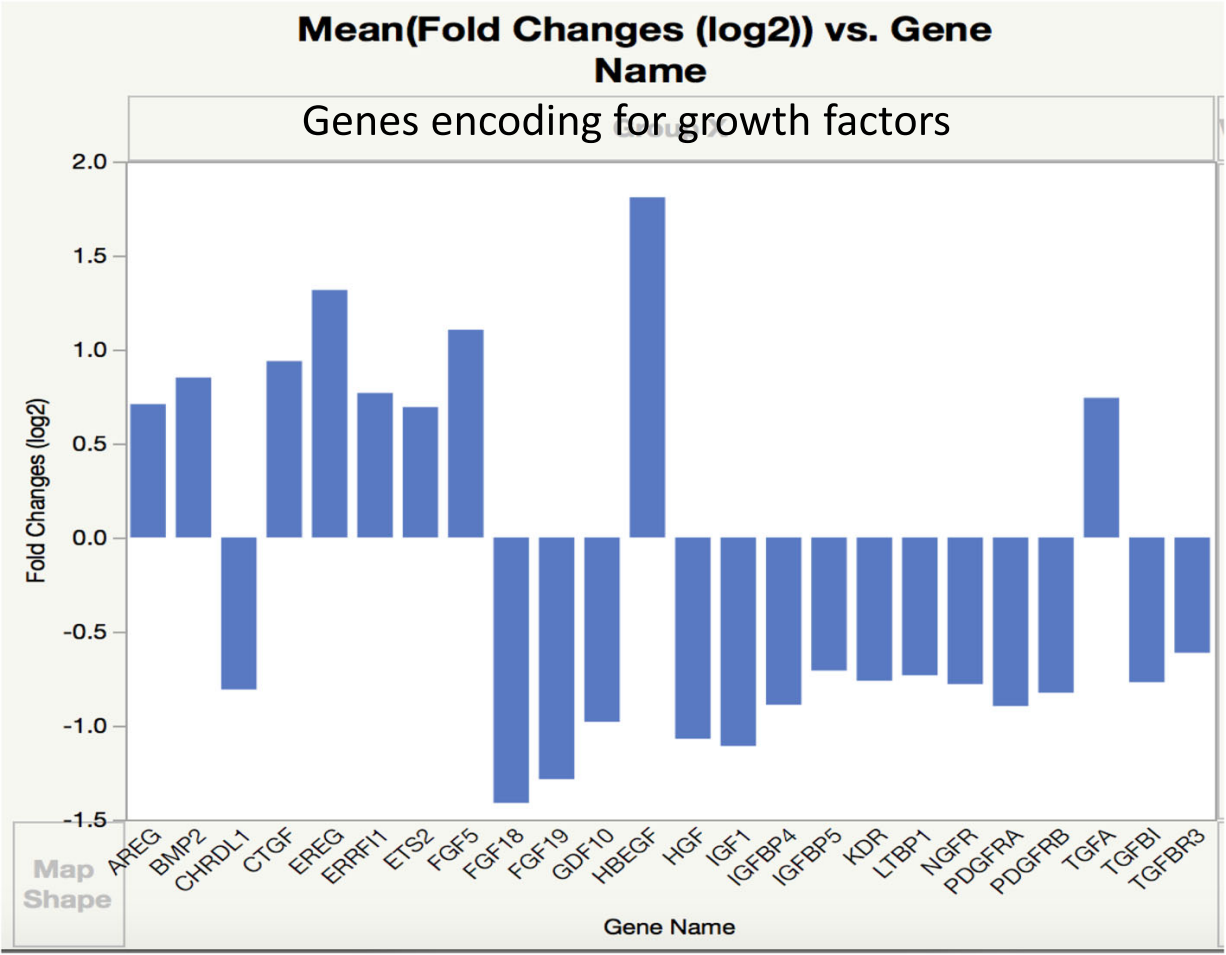
Expression of growth factor genes relevant to DSLD. Genes differentially expressed in DSLD were identified based on log fold change of 2

**Fig. 4:**
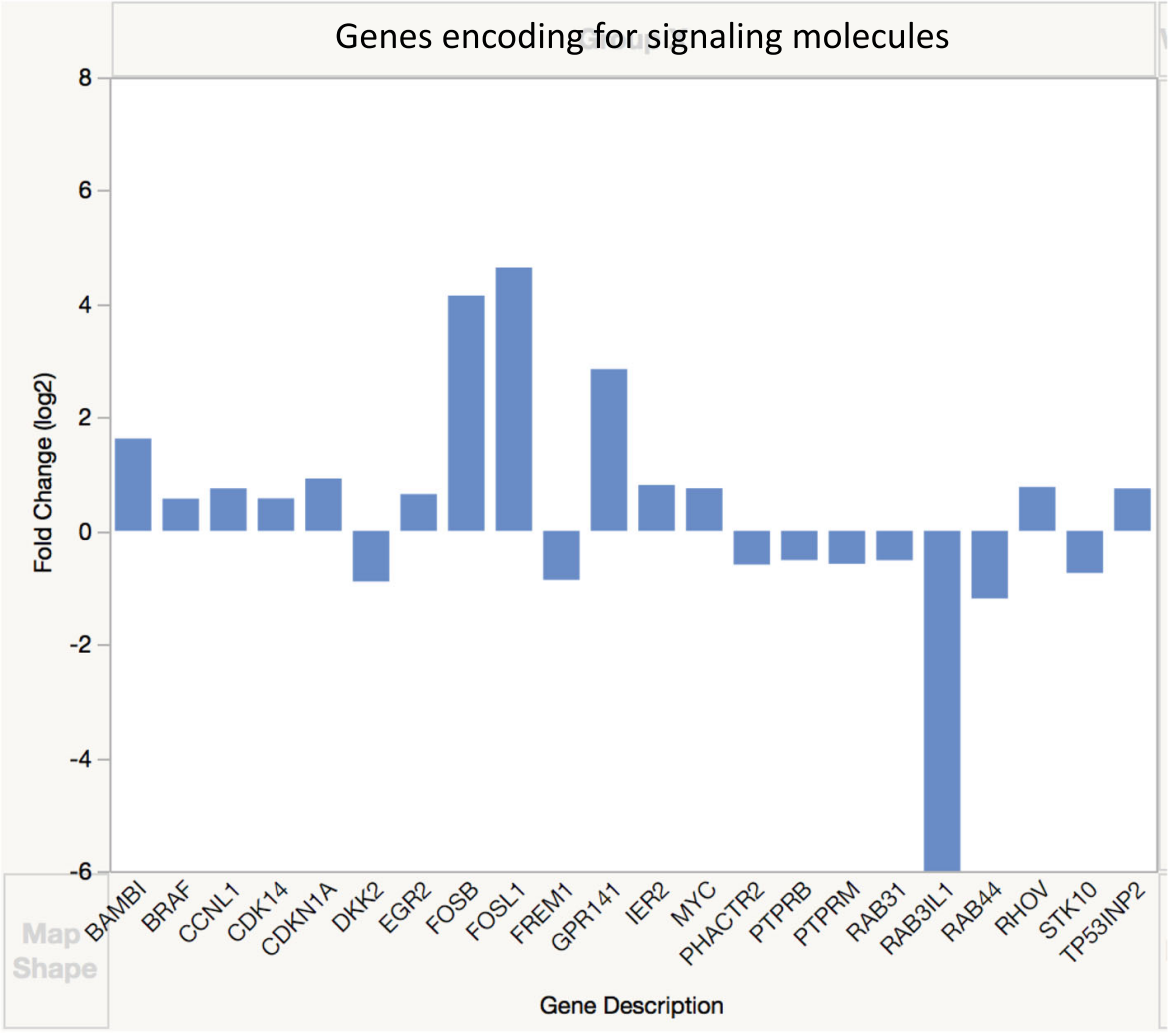
Expression of selected genes encoding for signaling mediators. Genes differentially expressed in DSLD were identified based on log fold change of 2

Several genes encoding for growth factors and their signaling mediators were overexpressed, other ones were underexpressed (Fig. 3). *FOS* was the most overexpressed gene from all 617 genes, perhaps because it is a transcription factor at the end of convergence of many signaling pathways of many growth factors. The overexpression of *BMP2*, though not very high, correlates well with our recent findings [8]. Though no expression changes were detected in the Smad signaling pathway, overexpression of genes encoding for many mediators and underexpression of some in the MAPK pathways was observed (Fig. 4, Supplemental data).

The genes for two chondrogenic growth factors, fibroblast growth factor (FGF)18 and 19, were downregulated. Another member of the FGF family, FGF5, was overexpressed.

Genes for several members of the epidermal growth factor (EGF) family were overexpressed (*HBEGF*, *EREG*, *TGFA*, *AREG*, and *ERRFI1*). Gene expression of connective tissue growth factor (CTGF), a mediator (but not a member of TGFβ family) of TGFβ activity was increased, whereas the expression of genes of some members of the TGFβ family itself was decreased (*TGFBR3*, *GDF10*—encoding for BMP3B, *LTBP1*—encoding for latent TGFβ-binding protein 1; *CHRDL1*; *TGFBI*). *CHRDL1* encodes chordin-like protein 1, an antagonist of BMP4 [20, 21]. Several members of the IGF family were underexpressed (*IGF1*, *IFGBP4*, and *IGFBP5*); this might be connected with BMP2 overexpression and is discussed into more details in the “Discussion” section. Genes for several angiogenic proteins, including PDGFRB, PDGFRA, dickkopf2, VEGFC, and KDR (VEGF receptor-kdr-like) [22] were underexpressed (Fig. 3, Supplemental data).

### Proteoglycans and relevant enzymes

**Fig. 5:**
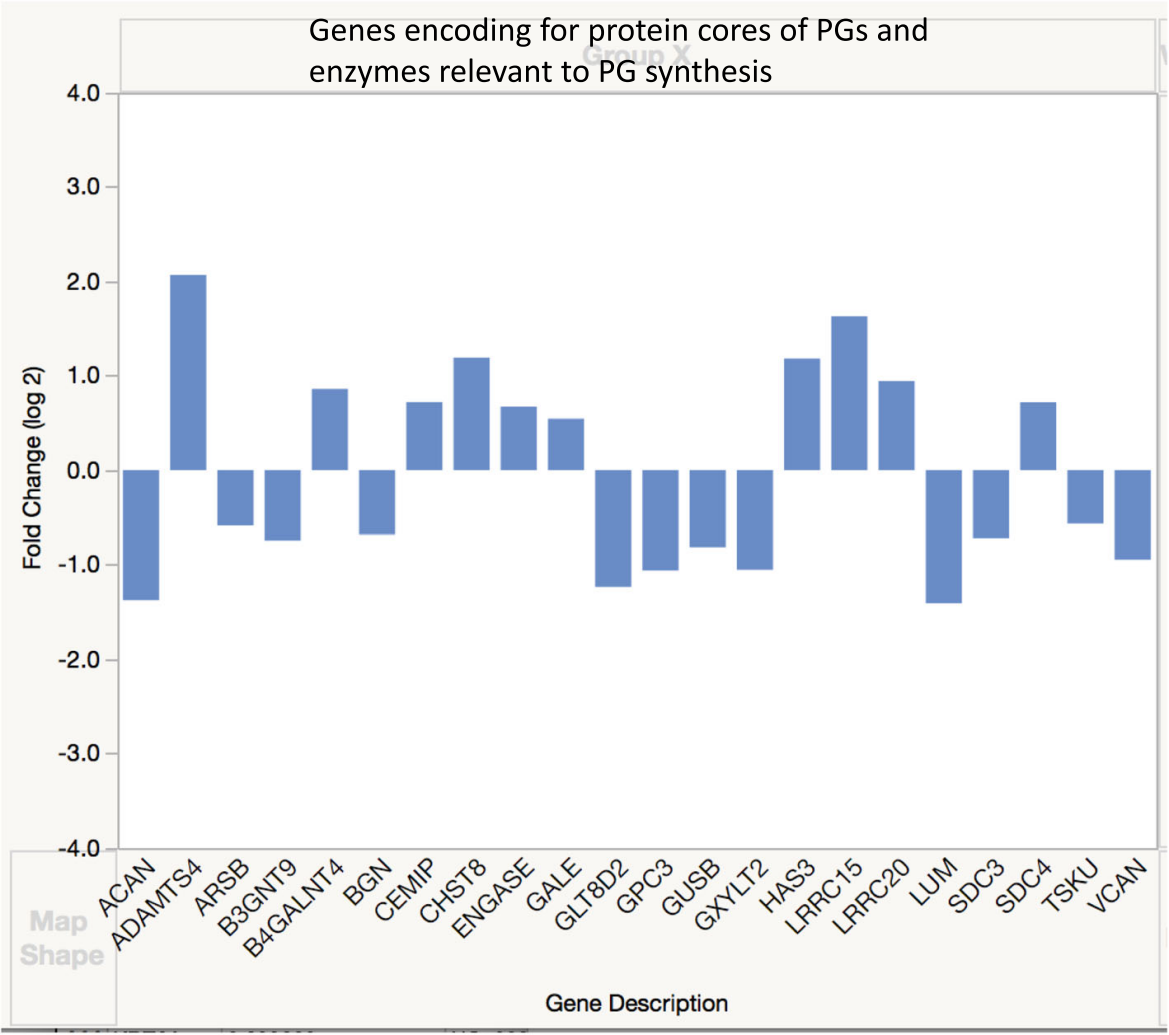
Expression of genes for core proteins of proteoglycans and related molecules. Genes differentially expressed in DSLD were identified based on log fold change of 2

The genes encoding for many protein cores of proteoglycans usually identified in tendon were underexpressed in DSLD, including many small leucine-rich proteoglycans (SLRPs), such as decorin, lumican, biglycan, and tsukushi (Fig. 5, Supplemental data). Even genes for protein cores of large proteoglycans, such *VCAN* encoding for versican, *ACAN* encoding for aggrecan, and *COMP* encoding for cartilage oligomeric matrix protein were underexpressed. In addition, the gene for ADAMTS4, also known as aggrecanase, was significantly upregulated in DSLD. Two genes for proteins relevant in hyaluronan synthesis, *HAS3* encoding for hyaluronan synthase 3 and *CEMIP* (encoding cell-migration inducing and hyaluronan-binding protein), were upregulated as well.

DEG was quite prominent among genes for many glycosyl transferases and other enzymes involved in synthesis and degradation of proteoglycans and glycoproteins. Some examples of underexpressed genes are *B3GNT8* (encodes for β-1,3-N acetylglucosaminotransferase 8, important for N-linked glycosylation, an enzyme regulating MMP2 and TIMP2), *SGSH* (encodes N-sulfoglucosamine sulfohydrolase, an enzyme degrading heparin sulfate, and likely playing a role in mucopolysaccharidoses, at least in people), *GLT8D2* (encoding glycosyltransferase 8 domain 2), and *B3GALT2* (beta-1-3-galactosyltransferase 2). Some overexpressed genes were identified as well, for example, *B4GALNT4* (encoding for beta-1-4-N-acetylgalactosaminyltransferase 4) and *CHST8* (encoding for carbohydrate sulfotransferase 8). A comprehensive list of all DEG related to proteoglycans and glycoproteins can be found in submitted Supplemental data.

### Collagens and other ECM components

**Fig. 6:**
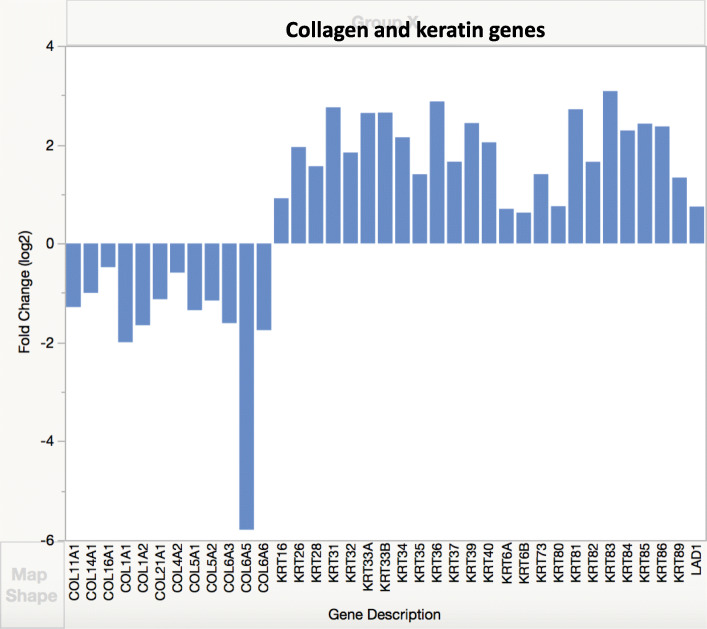
Expression of collagen and keratin genes. Genes differentially expressed in DSLD were identified based on log fold change of 2

Many genes encoding α chains of numerous collagen types were underexpressed with the exception of genes for α1 chains of types 26 and 17 collagens (Fig. 6, Supplemental data). Type 17 collagen regulates Wnt pathway and coordinates cell proliferation in interfollicular epidermis [23] and hair follicle stem cells [23, 24], and upregulates keratins.

**Fig. 7:**
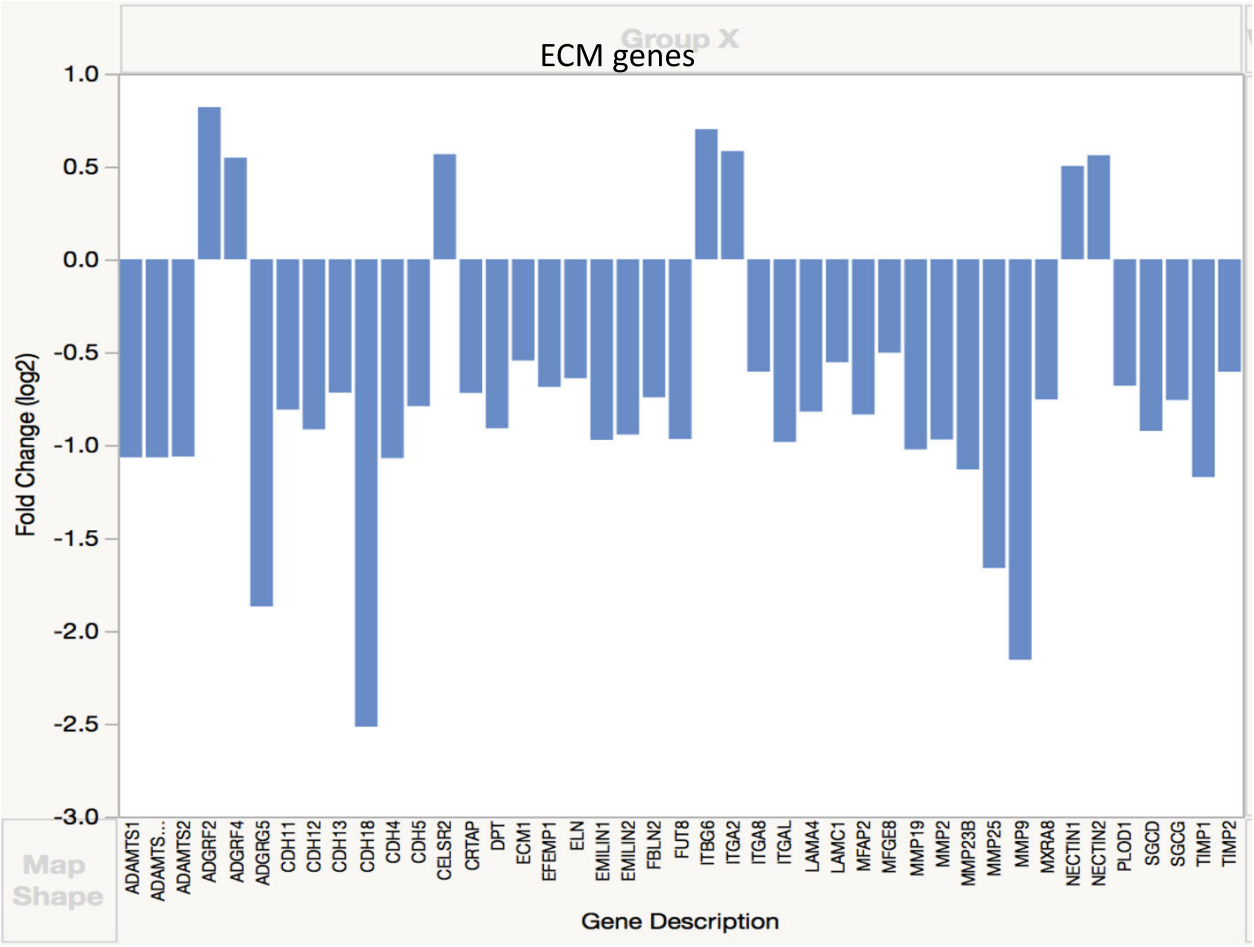
Expression of selected ECM genes. Genes differentially expressed in DSLD were identified based on log fold change of 2

Genes for several matrix metalloproteinases (MMPs 1, 9, 19, 23B, and 25) and at least two of the tissue inhibitors of metalloproteinases (TIMPs 1 and 2) were underexpressed (Fig. 7). Numerous other genes encoding many categories of ECM molecules were differentially expressed, including integrins, members of ADAMTS and ADAM families laminins, and fibulins (Fig. 7, see in Supplemental data).

### Keratins

Twenty eight epithelial and hair keratin genes of both types I and II [25] were upregulated, for some of them, the increase was statistically significant (Fig. 6, Supplemental data), in tandem with upregulated many genes for keratin-associated proteins as well.

### Immune function-related genes

**Fig. 8:**
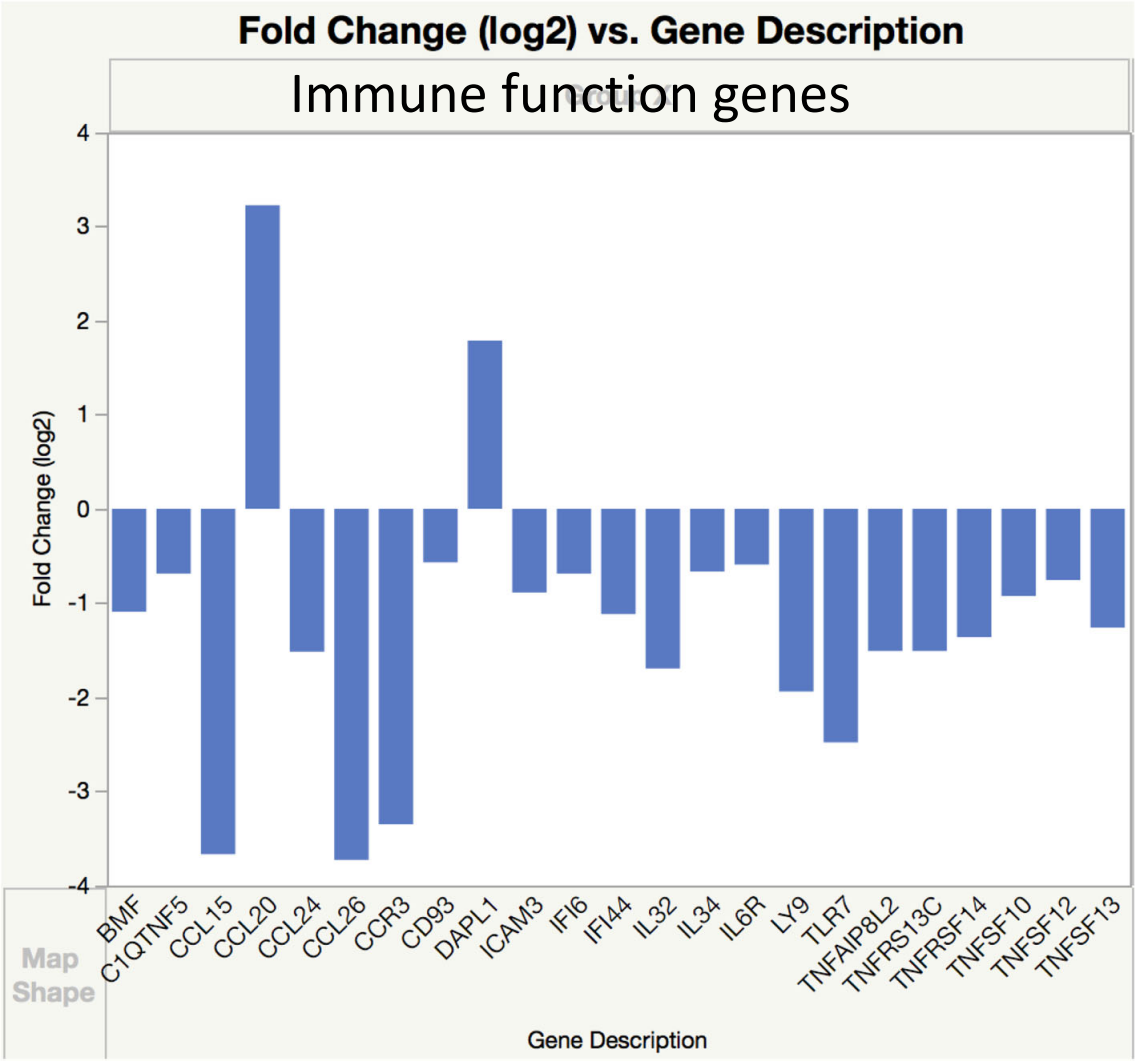
Expression of genes for selected molecules of immune function. Genes differentially expressed in DSLD were identified based on log fold change of 2

Expression of many cell-death-associated genes encoding for death-associated proteins (DAPs), including members of the tumor necrosis factor (TNF) system, both ligands and receptors was decreased (Fig. 8). For example, *TNFRSF13C*, *TNFAIP8L2*, *TNFRSF14*, *TNFSF10*, *TNFSF13*, *C1QTNF5*, and *TNFSF12* were in this group together with apoptosis-associated genes such as *BCL2*, *BAG2, BMF*, and *CD93*. An overall decrease was noticed in expression of genes for chemokines, interferons, and their receptors: *CCL24*, *CCL26*, *CCL15*, *CCR3, IFI6*, and *IFI44*. Gene expression was decreased for other proinflammatory cytokines as well: *IGSF10*, *IL34*, *IL6R*, *ICAM3*, *TLR7*, *IL32*, *LY9*. There was also decreased expression of genes for three members of a family of membrane-anchored enzymes, so called (a) disintegrin and metalloproteinases (ADAMs), 9, 19, and 33. Only a few pro-inflammatory molecules were overexpressed, among them *CCL20*, *IL17REL1*, and *IL18.*

Two genes encoding for corticosteroid 2 (*HSD11B2*) and for 3-β-hydroxysteroid dehydrogenase type 7 (*HSD3B7*), both regulating steroid metabolism, were underexpressed.

## Discussion

Our bioinformatics analysis of NGS and comparison of transcriptomes between RNAs of control and DSLD-affected horses provided a window into numerous factors and molecules potentially involved in DSLD. As skin biopsy would be preferred method as a source of a diagnostic marker, analysis of DEG in skin suggest the feasibility of using the results for development of a diagnostic assay for DSLD. This is comparable to the use of subcutaneous adipose-tissue derived fibroblasts rather than tendon-derived cells to study changes in gene expression in DSLD by Lu et al [26]. As shown by Seidler et al. and Miyake et al. cultures of skin fibroblasts have been a useful tool for determination of genetic and biochemical causes of several less common forms of Ehlers-Danlos syndrome, a systemic disease similar to DSLD [27, 28]. The good separation of control and DSLD transcriptomes into two clearly distinguishable groups suggests that a diagnostic assay based in these results could be developed and would validate our use of skin for RNA extraction. Moreover, DSLD-affected horses were clustered together regardless of their breed, Peruvian Pasos or warmbloods. We were not able to determine the role of age and sex on the DEG, in part because of the small number of horses in each group, and because of lack of more data on gene expression in horses in general. Peruvian Pasos are rather uncommon in the USA (about 5000 such horses are in the USA), and it was not easy to convince owners to participate in such study. Unfortunately, because horses are fairly long living and the course of DSLD is unpredictable and so far not examined in a systematic matter (e.g., prospective study), it is difficult to estimate the impact and course of RNA changes in the natural course of the disease. In addition, though we know that DSLD is a progressive disease, we do not know much about its pathogenesis and the nature of the progression. Ours is the first report examining DEG in DSLD.

We do recognize that this will have to be confirmed with a larger number of horses, both controls and with DSLD, and of other breeds as well. As expected, analysis of transcriptomes revealed differential expression in numerous genes, in 1567 to be exact, with more genes downregulated than upregulated. To no surprise, differential gene expression affected many proteoglycans, growth factors and signaling molecules, and ECM constituents. Our results brought some expected outcomes, and many unanticipated results as well.

As part of our ongoing efforts to identify a factor(s) initiating and/or driving the proteoglycan accumulation in DSLD, we reported on increased content of BMP2, a chondrogenic, osteogenic, and tenogenic growth factor and a member of the TGFβ super-family [29, 30], in cellular foci in DSLD. These foci consist of active fibroblasts/tenocytes with small amounts of proteoglycans and high content of BMP2 in their cytoplasm [8]. This finding correlates well with *BMP2* overexpression described here. The fact that skin RNA rather than RNA from tendon or ligament was analyzed may account for the relatively low degree of overexpression of *BMP2* in skin, and high overexpression of other, rather unexpected genes, such as those for keratins (see below). The *BMP2* overexpression was not accompanied by an increase in transcription in genes encoding for Smads, the mediators of the canonical TGFβ/BMP signaling pathways [31]. Instead, the observed overexpression of genes encoding for Fos and many mediators in the MAPK pathways indicates that MAPK pathway plays an important role in inappropriate expression and activity of BMP2 in DSLD. Crosstalk between TGFβ/BMP signaling and Ras/MAPK system has been noted in other systems as well [31–33].

The underexpression of *TGFB1*, *TGFBR3*, *LTBP1* (encoding for latent TGFβ-binding protein 1), *CHRDL1* (encoding for chordin-like 1 protein; antagonist of BMP4), and *TGFBI* (TGFβ induced), also known as *INHBA* (inhibin β A chain) genes correlates well with previously observed of no or only small changes in TGFβ content in DSLD [8].

Dysregulation of action of TGFβ and related molecules, such as BMPs (BMP2, BMP4, and BMP6) and CTGF (a mediator of BMP activity) in damaged tendons has been well documented in human and animal tendinopathies where the excessive presence of BMPs can lead to increased synthesis and deposition of proteoglycans in the tendon [29, 34]. *CTGF* (encoding for CTGF/CCN2) is active in chondrocytes and plays important roles in wound healing and fibrotic processes [35]. Under normal conditions, the regulation of terminal chondrocyte differentiation by CTGF/CCN2 is opposed by tsukushi, a member of the SLRP group, that affects proliferating and hypertrophic zones of the growth plate [36]. Underexpression of *TSK*, gene for tsukushi, might thus contribute to the presence of not well organized and differentiated cartilage islands in DSLD tendons [1].

Interestingly, both *FGF18* and *FGF19* genes, encoding for chondrogenic growth factors, were underexpressed, perhaps as the result of BMP2 overexpression. FGF18 is an anabolic chondrogenic and osteogenic growth factor acting through FGFR3 [37, 38]. We hypothesize that the underexpression of *FGF18* and *FGF19* may contribute to further underexpression of genes encoding for core proteins of many proteoglycans, especially those negatively regulated by BMP2 as well (Fig. 4). It is likely that *FGF5* overexpression is associated with overexpression of keratin genes (Fig. 6) as FGF5 is involved in normal follicle structure and hair growth [39].

Rui et al. have observed that treatment of tendon-derived stem cells with BMP2 leads to decrease in deposition of several proteoglycans, such as decorin, biglycan and fibromodulin, though they noted overall increase in GAG production and increase in aggrecan as well [30]. Obviously, the decrease in expression of many genes for core proteins of proteoglycans in DSLD tissues does remain somewhat mysterious as it is proteoglycans that accumulate in connective tissues in other organs besides tendons and ligaments in DSLD [1]. *ACAN*, gene encoding for aggrecan core protein, was downregulated, at least in skin, but *ADAMTS4* which encodes for aggrecanase was upregulated. The increase in *ADAMTS4* is in agreement with report by Plaas et al. [7]. However, they found an increased presence of aggrecan in DSLD-affected tendons and concluded that accelerated degradation of aggrecan by aggrecanases led to DSLD as the result of accumulation of aggrecan degradation products. Our previous, unpublished data found no changes in aggrecan staining in DSLD tendons. By the way, the degradation of articular cartilage in osteoarthritis is thought to be the results of ADAMTS5 and likely also of ADAMTS4 activity [38], two enzymes thought to be involved in degradation of certain SLRPs, e.g., of fibromodulin as well [40]. Though our previous work has demonstrated the presence of modified decorin in tendons with DSLD, it was clear from immunohistochemistry that the majority of the proteoglycan in these tissues was neither decorin nor aggrecan [1, 4].

The observed increased expression of hyaluronan synthase and binding protein genes may represent a compensatory mechanism of (attempted) increased hyaluronan synthesis which would offset the decrease in *ACAN* expression. This finding will have to be confirmed in other organs besides the skin. *TSK*, a gene encoding for tsukushi, a member of class IV SLRPs functionally related to class I SLRPs of which decorin and biglycan are also members [41] was underexpressed as well (see also above). Tsukushi, decorin, and biglycan are known to inhibit TGFβ/BMP/Smad pathways [42]. Several studies indicate that tsukushi modulates osteoblast differentiation through inhibition of BMP4 signaling, inhibits Wnt pathways, and regulates hair follicle cycle, all features it shares with decorin and biglycan [41, 42].

Gene defects in several human enzymes participating in GAG synthesis, among them xylosyltransferases 1 and 2, and at least two galactosyltransferases, are held responsible for several uncommon disorders affecting skeletal and joint structures [43]. A defect in B3GALT6 (encoding for β-1 l3-Galactosyltransferase-II) is tied to the progeroid type of Ehlers-Danlos syndrome [44]). We did report similarities between this type of Ehlers-Danlos syndrome and DSLD in our earlier work [4]. Some underexpressed and overexpressed genes encoding for enzymes involved in synthesis and degradation of proteoglycans and glycoproteins are listed in the “Results” section. We did not find any changes in the expression of glucuronyl C5-epimerase (dermatan sulfate epimerase), a limiting enzyme in the synthesis of dermatan sulfate [45]; however, the possibility of a mutation cannot be excluded. Previously, we hypothesized that this epimerase might play an important role in pathogenesis of DSLD [4]. A complete list of genes for enzymes of interest can be found in the submitted data set.

The expression of several other growth factors was decreased (Fig. 3). The significance of *GDF10* underexpression in DSLD is difficult to assess at this time. *GDF10* encodes for BMP3B. Though BMP3B was characterized as a primarily growth factor stimulating axonal sprouting in the cerebral cortex [46], it has been described also as an inhibitor of osteoblastic differentiation [47]. Similar phenomenon was observed with *IGF1*, and *IGFBP4* and *IGFBP5*. IGF-1 and IGFBP-4 are involved in stimulation of osteogenic differentiation. IGF-1 and IGFBP-4 promote proliferation and maturation of chondrocytes using the Wnt/catenin signaling pathway [48–50] whereas IGFBP-5 promotes fibrosis, cell senescence, and migration of macrophages, an inflammatory step preceding fibrosis [51, 52]. Whether the decrease in expression of IGF-1, IGFBP-4, and IGFBP-5 is the result of negative feedback by BMP2 or one of the other dysregulated growth factors or signaling molecules remains an open question. However, the lack of extensive calcifications in most cases of DSLD would be compatible with these results [1, 4, 53]. Primary calcifying desmopathy in horses presents as extensive calcifications of tendons but it is encountered rather infrequently [54]. The underexpression of several members of the PDGF/VEGF family (*VEGFC*, *KDR* - encoding for VEGF receptor-kdr-like protein, *PDGFRB*, and *PDGFRA*) is more difficult to explain as their expression is enhanced in other systems by increased BMP2 presence [29]; however, this corresponds to minimal presence of significant blood vessels in the DSLD-affected tissues, including active foci producing BMP2 [1, 8].

Genes encoding α chains of numerous collagen types were underexpressed. This is indicative of profound disturbance in collagen metabolism, whether it is the consequence of altered expression of BMP2 or changes in proteoglycan synthesis remains to be established [29, 55].

Only genes for α chains of two collagen types were overexpressed, one for the α chain for type 17 collagen, the 2^nd^ gene was for the α chain for type 26 collagen. Type 17 collagen coordinates cell proliferation in interfollicular epidermis [23]. Its function was shown to be defective in human epidermolysis bullosa [24]. It is possible that its overexpression in DSLD horses explains the presence of loose wrinkly skin, patches of gray hair, and bruises in some of these horses (personal communications). In addition, the overexpression of BMP2 may contribute to these changes as well. BMP2 plays a significant role in the embryonic development of skin and its appendages, including hair follicles, specifically in hair placode [56], whereas BMP4 directs the development in mesenchymal cells located beneath the hair placode [57]. A more recent report has shown that overexpression of constitutively active BMP-receptor-IB (one of the receptors for BMP2) in transgenic mice leads to ichthyosis-vulgaris-like skin disorder characterized by hyperkeratosis [58]. The overexpression of *FGF5* in DSLD transcriptomes points to a possible involvement of FGF5 in impaired hair growth [39, 59]

Phenotypically, DSLD is clearly and unequivocally distinct from Hereditary Equine Regional Dermal Asthenia (HERDA) [60, 61] and Warmblood fragile Foal syndrome or WFFS with primary skin involvement, and only occasional presence of affected tendons and joints [62]. A pinpoint mutation in the equine procollagen-lysine, 2-oxoglutarate 5-dioxygenase (*PLOD1*) gene is implicated as the cause of WFFS, an autosomal recessive condition. Horses affected with WFFS present shortly after birth with thin fragile skin, hyperextended joints, and poor wound healing [62]. People with mutation in *PLOD1* suffer from so called kyphoscoliotic Ehlers-Danlos syndrome [63], a disorder reminiscent of other, rare types of Ehlers-Danlos with mutations in carbonic sulfotransferase 14 or dermatan-sulfate epimerase [64].

The role and significance of type 26 collagen is unknown. Its expression appears to be limited to the testis and ovary [65].

Genes for MMPs 1, 9, 19, 23B, and 25 and at least two of the tissue inhibitors of metalloproteinases (TIMPs 1 and 2) were underexpressed. TIMP1 inhibits the activity of MMP 9 [66], and it is thought that TIMP1 plays an important role in limiting inflammation following injury [67]. TIMP2 inhibits the activity of MMP2, but it is also participatory in indirect activation of MMP2 through association with MMP14 that may promote cancer progression [68] and, more importantly in the context of DSLD, aortic aneurysm development [69]. It might be of some significance that not only these MMPs are collagenases and/or gelatinases, but most of them degrade proteoglycans (e.g., aggrecan and versican) as well [66, 68].

Hofberger et al. have associated idiopathic chronic degeneration of the SL, including DSLD, with pituitary pars intermedia dysfunction or PPID [70, 71]. PPID is characterized by elevated free cortisol fraction levels accompanied by increased immunostaining for 11-β-dehydrogenase type I in SL and skin. We did not notice any changes in expression of *HSD11B1* gene (which encodes for 11-β-dehydrogenase type I); however, *HSD11B2* gene encoding for 11-β-dehydrogenase type II was found to be underexpressed. Similar decrease in staining for 11-β-dehydrogenase type II was predicted, but not verified by Hofberger et al. [71]. Interestingly, they did find skin thinning in their PPID-affected horses. Whether the decrease in gene for 11-β-dehydrogenase type II, and SL and skin changes in horse with DSLD found by us are analogous to findings identified in horses with PPID by Hofberger remains to be determined. No clinical signs of PPID were observed by us, owners, and any of the veterinarians who provided skin samples or horses for our study.

The lack of inflammatory cells in DSLD-affected tissues is rather conspicuous [1, 8]. As noted in the “Results” section, many genes for proinflammatory proteins and peptides, including chemokines, TNF-α, and TNF-α-system-related molecules, were downregulated. The expression of genes for ADAM 9, 19, and 33 was decreased as well. In general, ADAM genes and their products are involved in a variety of pro-inflammatory processes. ADAM 9 and 19 are membrane-anchored enzymes activating cytokine precursors, including that for TNF-α into active molecules [72, 73]. *ADAM 33*, the third underexpressed gene of the ADAM family, has been identified as a susceptibility gene for asthma and chronic obstructive pulmonary disease, and it likely plays a role in stimulating immune function, and remodeling of extracellular matrix [74].

Though NGS is a powerful tool to evaluate level of expression of individual genes or transcriptomes, it does not tell us much about the translation mRNAs into actual protein synthesis and function. Another drawback of NGS is that it does not identify the presence of mutations in individual genes that might be instrumental in pathogenesis of DSLD, more specifically, in the increased proteoglycan presence either due to a mutation in a core protein of a less characterized proteoglycan, or in an enzyme facilitating synthesis of GAGs attached to proteoglycans.

## Conclusions

Our study of changes in skin transcriptomes in equine DSLD confirms our previous findings that strongly indicated that DSLD is a systemic disorder characterized by disturbances of components of extracellular matrix, such as proteoglycans. The decreased expression of genes for numerous protein cores of proteoglycans and several genes for enzymes responsible for proper synthesis of GAG chains was identified. The decreased expression of genes for collagen α chains indicates more global disruption of extracellular matrix metabolism. The increased expression of hyaluronan synthase and binding protein genes described in this study may represent a compensatory mechanism of increased hyaluronan synthesis which would offset the decrease in *ACAN* expression, and be responsible for at least partially inappropriate accumulation of proteoglycan material in ECM of DSLD-affected tissues. The increased *BMP2* gene expression support previous finding of increased presence of BMP2, a chondrogenic member of the TGFβ family, and may explain, together with decreased *FGF18* and *FGF19* expression why we found disordered proteoglycan expression. The use of skin tissues rather than tendon tissue for NGS explains the rather prominent overexpression for keratins. Though skin may have different pattern of gene expression than tendons, it does contain connective tissue and this is reflected in several aspects of the DEG pattern in skin. Moreover, a differential expression of certain genes, such as genes for keratins, in DSLD skin may clear a path for development of a specific diagnostic test utilizing skin as an accessible source of a biomarker.

## Supplementary information

**Additional file 1.** Halper Cufflinks gene DEG results.

## Data Availability

The raw sequence data have been deposited in the NCBI Sequence Read Archive (accession number: PRJNA544650).

## Abbreviations

ADAM: A disintegrin and metalloproteinase
ADAMTS: A disintegrin and metalloproteinase with thrombospondin motifs
BMP: Bone morphogenetic protein
CTGF: Connective tissue growth factor
DAP: Death-associated protein
DEGs: Differentially expressed genes
DSLD: Degenerative suspensory ligament desmitis
ECM: Extracellular matrix
EGF: Epidermal growth factor
FGF: Fibroblast growth factor
FGFR3: FGF receptor 3
GAG: Glycosaminoglycan
HERDA: Hereditary Equine Regional Dermal Asthenia
MMP: matrix metalloproteinase
KDR: VEGF receptor-kdr-like
INF-γ: Interferon-gamma
*LTBP1*: (Gene for) latent TGFβ-binding protein
NGS: Next generation sequencing
PCA: Principal component analysis
PDGFRA: Platelet-derived growth factor receptor A
PG: Proteoglycan
PPID: Pituitary pars intermedia dysfunction
TGF: Transforming growth factor
TGFβR: TGFβ receptor
TIMP: Tissue inhibitor of metalloproteinase
TNF: Tumor necrosis factor
WFFS: Warmblood fragile foal syndrome
